# Pediatric Integrative Medicine: Vision for the Future

**DOI:** 10.3390/children5080111

**Published:** 2018-08-20

**Authors:** Anna Esparham, Sanghamitra Misra, Erica Sibinga, Timothy Culbert, Kathi Kemper, Hilary McClafferty, Sunita Vohra, Lawrence Rosen

**Affiliations:** 1 Division of Child Neurology-Headache Section, Children’s Mercy Hospital, University of Missouri School of Medicine-Kansas City, Kansas City, MO 64108, USA; 2 Mobile Clinic Program, Texas Children’s Hospital, Baylor College of Medicine, Houston, TX 77054, USA; smisra@bcm.edu; 3 Department of Pediatrics, Johns Hopkins School of Medicine, Baltimore, MD 21205, USA; esibinga@jhmi.edu; 4 Integrative Medicine, Prairie Care, University of Minnesota Medical School, Chaska, MN 55318, USA; tculbert@prairie-care.com; 5 Department of Pediatrics, College of Medicine, Ohio State University, Columbus, OH 43210, USA; kathi.kemper@osumc.edu; 6 Department of Medicine, Arizona Center for Integrative Medicine, University of Arizona, Tucson, AZ 85724, USA; hmcclafferty@email.arizona.edu; 7 Integrative Health Institute, CARE Program, PedCAM Network, Department of Pediatrics, Medicine, and Psychiatry, Faculty of Medicine and Dentistry, University of Alberta, Edmonton, AB T6G 2C8, Canada; svohra@ualberta.ca; 8 Whole Child Center, Oradell, NJ 07649, USA; ldrdoc@alum.mit.edu

**Keywords:** pediatric integrative medicine, vision, clinical practice, education, advocacy, complementary therapies

## Abstract

Pediatric integrative medicine (PIM) is of significant interest to patients, with 12% of the general pediatric population and up to 80% of children with chronic conditions using PIM approaches. The field of PIM has evolved over the past 25 years, approaching child health with a number of guiding principles: preventive, context-centered, relationship-based, personalized, participatory, and ecologically sustainable. This manuscript reviews important time points for the field of PIM and reports on a series of meetings of PIM leaders, aimed at assessing the state of the field and planning for its future. Efforts in the first decade of the 2000s led to increased visibility in academic and professional pediatric organizations and through international listservs, designed to link those interested in and practicing PIM, all of which continue to flourish. The PIM leadership summits in recent years resulted in specific goals to advance PIM further in the following key areas: research, clinical practice, professional education, patient and family education, and advocacy and partnerships. Additionally, goals were developed for greater expansion of PIM professional education, broader support for pediatric PIM research, and an expanded role for PIM approaches in the provision of pediatric care.

## 1. Introduction

Integrative medicine is defined as relationship-centered care that focuses on the whole person, is informed by evidence, and makes use of all appropriate therapeutic approaches, healthcare professionals and disciplines to achieve optimal health and healing, including evidence-based complementary and alternative medicine. Pediatric integrative medicine (PIM) develops and promotes this approach within the field of pediatrics [1,2].

The field of pediatrics is at a crossroads. The health of our children—our future—is at stake. The prevalence rates of myriad chronic pediatric health conditions continue to rise at an unprecedented pace [3,4]. Chronic health conditions contribute to the global burden of disability [5]. Historically, the conventional solution has favored a ‘disease-treatment system’ that often incentivizes more invasive care at higher cost, an approach poorly aligned with the needs of today’s children. Health care transformation is no longer optional; it is an absolute imperative. Advocates and stakeholders in search of better health care require new approaches to meet the complex health challenges faced by children. We are searching for safer and more cost-effective paradigms to optimize the health and well-being of children everywhere. Multidisciplinary healthcare models that implement integrative and complementary therapies for patients with chronic illness have shown improved clinical outcomes and quality of life [6,7]. In addition, health outcomes are improved through patient empowerment [8,9]. Integrative medicine promotes relationship-centered care and empowers individuals to incorporate wellness strategies such as improving nutrition, physical activity, and sleep hygiene.

Pediatric integrative medicine (PIM) represents an evolution in pediatric care, a paradigm that embodies a philosophy consistent with long-standing holistic principles of quality medical care. As Dr. Kathi Kemper noted in her Presidential Address to the Ambulatory Pediatric Association nearly 20 years ago, “Holistic medicine is really just good medicine. It means caring for the whole child in the context of that child’s values, their family’s beliefs, their family system, and their culture in the larger community, and considering a range of therapies based on the evidence of their benefits and cost” [10] (p. 214).

**Figure 1:**
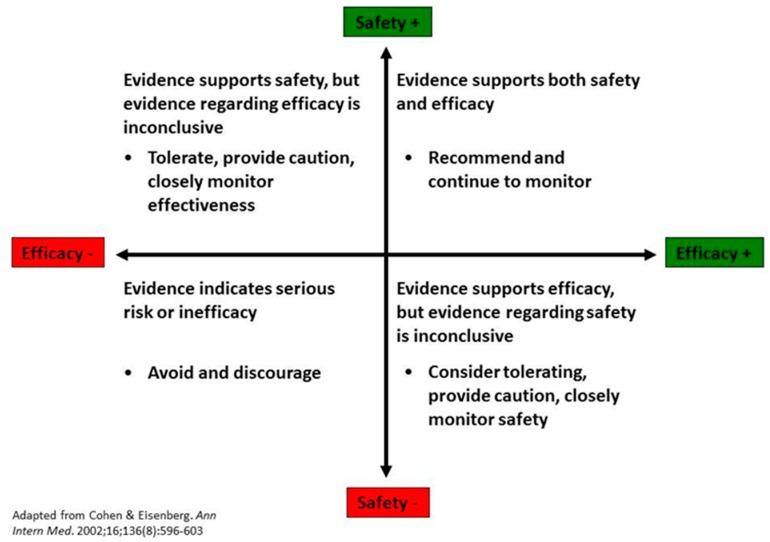
Safety–effectiveness therapy evaluation rubric.

PIM is defined by several core guiding principles [2,11]:

- Preventive: True primary care pediatrics is proactive rather than reactive. Prescribing lifestyle solutions to prevent disease is generally preferable to costly and potentially risky treatments. Lifestyle prescriptions may include food, activity, nature, creativity, rest, mindfulness, and connection with others.
- Context-centered: Children must be nurtured within the context of healthy families, communities, and schools. Health in mind, body, and spirit depends on how suitable the environment is for the child.
- Relationship-based: Only through open communication and building trust are we best able to work together to ensure each child’s optimal health. The connection between health professionals and families has its own healing potential.
- Personalized: Health is not a one-size-fits-all proposition. Each child carries a unique potential based on a complex interplay of genetic and environmental factors. There is no medical treatment that can be guaranteed as safe for 100% of any population. Each family has the inherent right to make health care decisions for their children, keeping in mind the best interests of the child as well as legitimate public health concerns that ethically inform these decisions.
- Participatory: Creating health should be a collaborative process, actively encouraging participation and putting children and families back in control of their own health. Patient-centered care creates hope and empowers families to make sustainable changes, inspiring children to create the future they deserve.
- Ecologically sustainable: How we practice healthcare affects the environment, which has a measurable and cyclical impact on our health. The health and well-being of all the Earth’s inhabitants are intimately tied to the health of our planet.
- Evidence-informed: Therapies that are evidence-informed while using the safety-effectiveness rubric (Figure 1) are considered as part of the treatment plan.

## 2. Background

The field of PIM has developed organically over the last 25 years owing to pressing need and interest from pediatric health professionals, parents, and children. We have clear and compelling data that complementary therapies are of great interest to parents [12]. Use of these therapies in pediatrics is significant, as the 2012 National Health Interview Survey revealed that 12% of children use complementary therapies [13]. Studies in specific chronically ill populations have reported complementary therapy use in up to 80% of study participants [14,15]. We use the term “complementary” specifically to delineate these therapies from those offered within conventional Western medical care systems. PIM, by definition, embodies the integration of complementary therapies with conventional pediatric health care in a way that is informed by best available evidence and centered on the individual patient and family. Truly effective and safe patient-centered care demands that we talk with our patients about therapies of interest to them and know where to find evidence to inform these conversations.

Critics maintain that integrative therapies do not have sufficient evidence to support their use in pediatric populations. We recognize that all health interventions would benefit from more high quality pediatric research to assess both effectiveness and safety. We do not agree that, by definition, all conventional care is evidence-based and that complementary therapies, by definition, lack evidence, or else they would be incorporated as part of conventional care. Research published in reputable journals has shown over the last 30 years that myriad complementary therapies are safe and effective for a variety of pediatric conditions and symptoms. Pediatric health professionals are encouraged to ask their patients and families about complementary therapy use [12]. In a national survey sent to members of the American Academy of Pediatrics (AAP), most pediatric healthcare professionals (87%) had been asked about complementary and alternative (CAM) therapies by a patient/parent within three months of the survey [16]. In addition, over 80% of the pediatric healthcare professionals desired additional information on CAM, with fewer than 5% reporting being knowledgeable about individual CAM therapies [16]. Of the whether or not professionals ‘believe’ in these therapies is immaterial; patient-centered care and patient safety demand that professionals know what health approaches are being used and considered. Health professionals must remember that evidence-informed medicine is a triad: best available evidence, clinical experience, and patient preference [17]. Dr. Kathi Kemper and Mr. Cohen, JD, MBA, MFA published a now well-established rubric to evaluate the use of therapies [18], see Figure 1.

As demonstrated in the graphic, use of therapies should be recommended if the intervention is known to be safe and effective based on best available evidence. If the therapy is known to be safe but efficacy has not yet been conclusively established, it is reasonable to tolerate use while monitoring response. If a therapy is known to be effective but its safety is questionable, after a careful informed consent discussion, use may be considered if monitored closely. Finally, a therapy is to be avoided and discouraged if it has no proven efficacy and known adverse effects. Relative risks and benefits should always be considered in light of best available evidence, and professionals can use the graphic to help guide decision-making in collaboration with patients and families. It is important to note that this rubric applies to all therapies—not just those deemed complementary—as complementary therapies may not apply to all pediatric conditions.

We recognize there are pediatric health professionals who would like to know how to discuss and apply these therapies in clinical practice, where to seek further training, and when to refer patients for these therapies. In addition, several barriers may exist for both professionals and families, such as adequate coverage of therapies and availability of PIM resources for the underserved or for those living in resource-poor locations [19,20]. The PIM Leadership Initiative (PIMLI) has developed this position statement to provide a unified vision for PIM to meet the needs of professionals in the service of pediatric patients and families. As PIM is still growing, although 20 years old, providing structure and vision is imperative for the continued, sustainable development of the field.

## 3. Evolution of Pediatric Integrative Medicine

The field of PIM has evolved tremendously over the past two decades. The sentinel events that have shaped the current PIM landscape are noted below.

Pediatric integrative medicine timeline, 1995–2017:

- 1995 Ambulatory Pediatric Association establishes Holistic Pediatrics Special Interest Group
- 1996 “The Holistic Pediatrician” published (Kemper)
- 1998 Boston Children’s Hospital establishes Center for Holistic Pediatric Education and Research
- 1999 First PIM Conference hosted by University of Arizona; Dr. Kemper’s APA Presidential Address (*Holistic Pediatrics = Good Medicine*)
- 2000 Second PIM Conference hosted by Children’s Hospitals and Clinics (Minneapolis) and University of Minnesota, AAP Task Force on CAM established and AAP NCE CAM sessions start
- 2004 First PIMLI Summit in St. Paul, MN; Pediatric Complementary and Alternative Medicine Research & Education (PedCAM) Network (Vohra) and International Pediatric Integrative Medicine (IPIM) Network (Rosen) started
- 2005 AAP Provisional Section on Complementary, Holistic, and Integrative Medicine (CHIM) and Integrative Pediatrics Council (IPC) established
- 2005–2008 International PIM/Pangea conferences hosted by IPC
- 2006 *Pediatrics in Review* series on PIM debuts (Vohra, editor)
- 2007 *Pediatric Clinics of North America* volume on PIM published (Rosen, Riley ed.)
- 2008 AAP Section on CHIM official (name later changed to Section on Complementary and Integrative Medicine, or SOCIM); *The Use of Complementary and Alternative Medicine in Pediatrics* AAP Clinical Report published in *Pediatrics*, IPC dissolves
- 2009 “Textbook on Integrative Pediatrics” published (Culbert, Olness ed.)
- 2012 PIM program survey published (*Pediatric integrative medicine: pediatrics’ newest subspecialty?* Vohra S, et al.); University of Arizona Center for Integrative Medicine launches Pediatric Integrative Medicine in Residency (PIMR) curriculum; AAP SOCIM name changed to Section on Integrative Medicine (SOIM)
- 2014 *Physician Health and Wellness* AAP Clinical Report published in *Pediatrics*; inaugural AAP SOIM Pioneer Award given to Dr. Kemper; “A Guide to Integrative Pediatrics” textbook published (Misra, Verissimo)
- 2015 PIMLI Summit II in Boston, MA
- 2016 PIMLI Summit III in Hackensack, NJ; “The Holistic Pediatrician, 20th Anniversary Edition” published (Kemper); *Mind-Body Therapies in Children and Youth* AAP Clinical Report published in *Pediatrics*
- 2017 “Integrative Pediatrics: Art, Science, and Clinical Application” textbook published (McClafferty)

All events are listed to the best of the PIMLI’s Summit collective knowledge and may not include all international PIM events.

As noted, the first official meeting of PIM leaders in the United States was held in 2004 and termed the Pediatric Integrative Medicine Leadership Initiative (PIMLI) Summit. The first PIMLI Summit spawned several major initiatives: A Section on Integrative Medicine within the AAP, an interprofessional PIM nonprofit (the IPC), and two international online resources (the IPIM-Network and PedCAM). These entities spearheaded numerous educational programs, including (1) PIM content at the AAP’s National Conferences and Pangea PIM Conferences; (2) publication of key PIM books and scholarly articles; and (3) the establishment of the PIM in Residency (PIMR) training program. The decade of significant progress following this first PIMLI Summit coupled with the desire to nurture sustainable growth in the field prompted PIM leaders to systemically revisit the vision for the future of PIM [2,21,22].

## 4. 2015 PIMLI Summit

Supported by a generous grant from the Marino Health Foundation, Drs. Kathi Kemper and Lawrence Rosen—in partnership with the AAP—developed the second PIMLI Summit held in Boston, MA in June 2015 to accomplish the following objectives: (1) develop a long-term vision for PIM addressing gaps and future needs for clinical, educational, research, and advocacy domains; and (2) develop future PIM leaders who can achieve the vision through practical strategies and partnerships.

A group of fifteen PIM leaders from the United States and Canada were invited to attend the Summit. These physicians had a varying range of expertise in PIM with different stages of career development, and practiced in a variety of settings. They also represented the AAP Section on Integrative Medicine, Academy of Integrative Health and Medicine, University of Arizona Pediatric Integrative Medicine in Residency program, various North American pediatric academic programs, and the PedCAM and IPIM Networks. An intentional effort was made to incorporate a balance of early career and established leaders. Representatives from many other professions and partner organizations were consulted during meeting preparation to optimize long-term sustainability and success and to embody the interprofessional philosophy inherent in integrative care. Specifically, with organizational support by John Weeks, a pioneer in collaborative interprofessional IM efforts over several decades, representatives from the Academic Consortium for Integrative Medicine and Health (ACIMH) participated in a comprehensive pre-Summit survey.

### 4.1. Professional Pre-Summit Survey Summary

**Figure 2:**
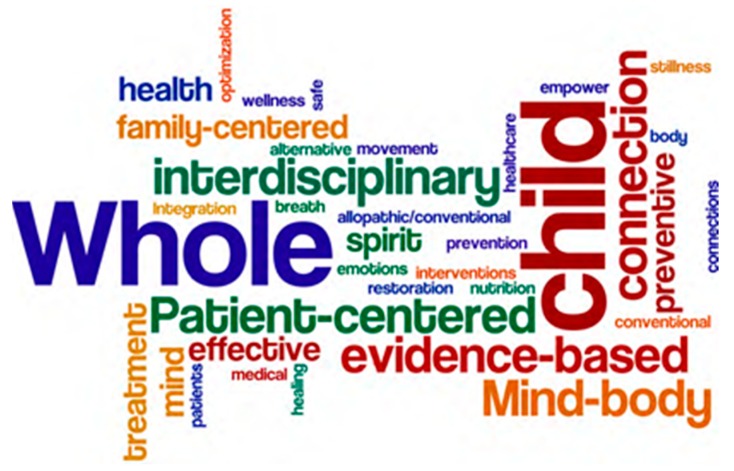
What does integrative medicine mean to you?

In May 2015, more than 30 integrative health professionals provided feedback on the definition of integrative medicine, the state of child health, and a vision for PIM through a pre-Summit survey developed by Drs. Kemper and Rosen (unpublished data). In addition to pediatricians, these professionals represented expertise in Ayurvedic Medicine, botanical medicine, chiropractic, education, energy medicine, family medicine, health policy, massage therapy, mental health, naturopathy, Traditional Chinese Medicine, and yoga. Drs. Kemper and Rosen coordinated a conference call with John Weeks and a specially-designed 15-member interprofessional task force to discuss the survey and provide guidance for the Summit. A wordcloud (Figure 2) was created from stakeholder comments about what integrative medicine meant to them (unpublished data).

Regarding the current state of children’s health, respondents noted that while pediatric acute care is ‘good’, both the quality and quantity of medical care for children and youth with chronic conditions often is not optimal. Survey participants felt the current conventional health care system is failing to adequately address the rising prevalence of chronic illnesses in children. They believed there is a need for a larger workforce of PIM health professionals to meet the challenges of complex childhood conditions while also promoting wellness. They also highlighted the importance of educating families about lifestyle approaches including healthy nutrition, physical activity, sleep, and stress coping skills. A need for more effective advocacy for improving children’s health globally was mentioned, including a push for healthier modes of transportation, sustainable agriculture and food sources, and addressing health related environmental factors.

When asked how pediatricians can more effectively work with other stakeholders to best serve children’s needs, survey participants responded that collaboration with other health professionals, parent groups, third-party payers, and community organizations is key. PIM stakeholders’ vision for what pediatric integrative medicine would look like in 10 years included the wish that “decisions regarding a child’s healthcare would be made from an integrative perspective, taking all possible healthcare options into account”. Pediatric clinics would provide integrative care as part of the medical home with respect for all members of the patient’s health care team. In addition, schools that train health professionals will incorporate integrative medicine training and provide interprofessional learning environments to build trust and facilitate patient-centered collaborative care. Finally, increased public and private funding would be available to support the investigation of all evidence-informed approaches that are personalized to the individual, given their health, preferences, and goals.

### 4.2. Parent Survey Summary

**Figure 3:**
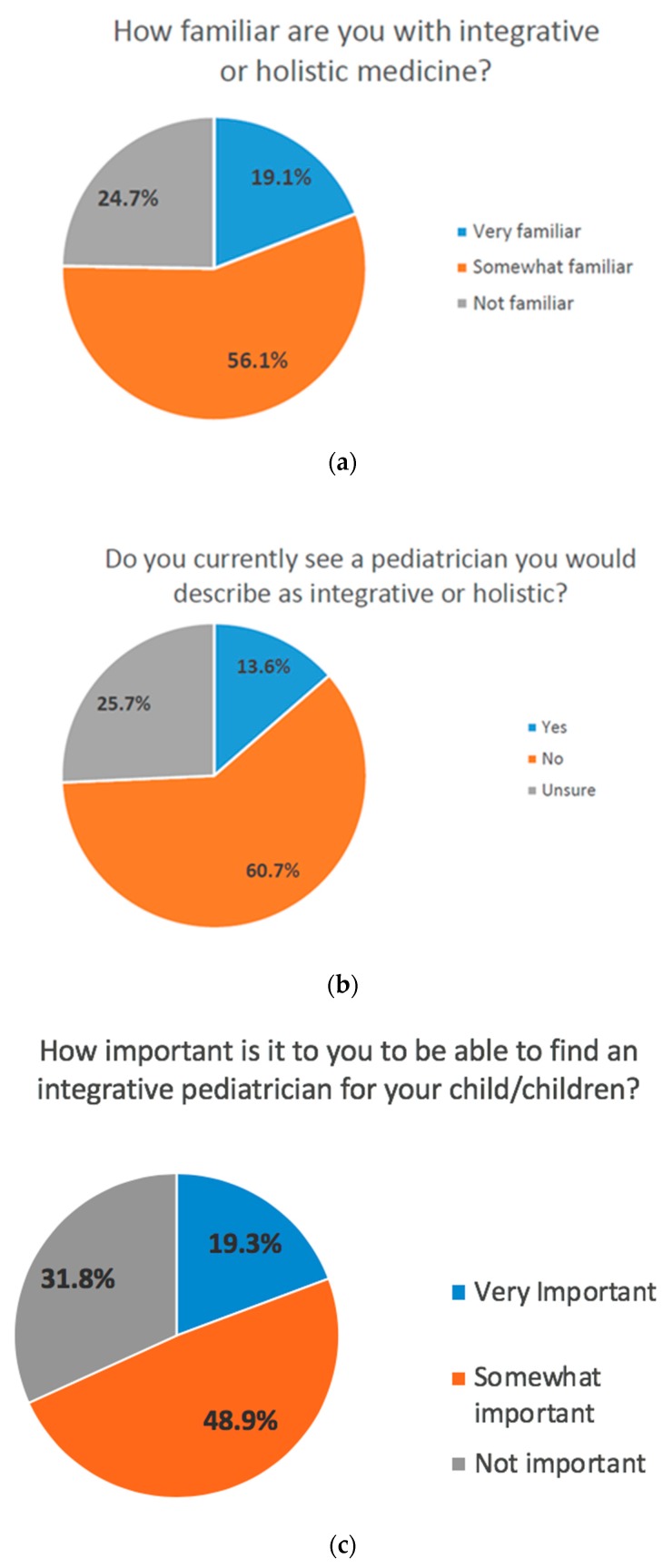

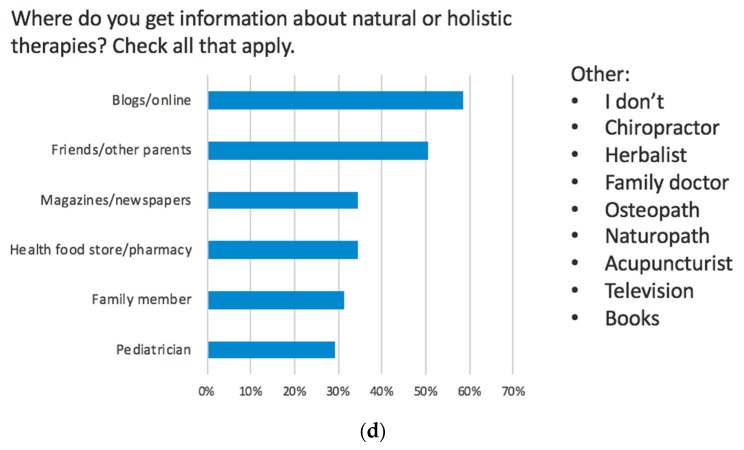
(**a**) Parent response to question “how familiar are you with integrative or holistic medicine?” Answer options included: very familiar, somewhat familiar, and not familiar; (**b**) Parent response to question “do you currently see a pediatrician you would describe as integrative or holistic?” Answer options included: yes, no, and unsure; (**c**) Parent response to question “how important is it to you to be able to find an integrative pediatrician for your child/children?” Answer options included: very important, somewhat important, and not important; (**d**) Parent response to question “where do you get information about natural or holistic therapies?” Answer options included all options that applied to parents: blogs/websites, friends/other parents, magazines/newspapers, health food store/pharmacy, family member, pediatrician, and other (as outlined above).

Parents are key PIM stakeholders and, therefore, a national parent online survey was conducted just prior to the PIMLI Summit to guide the meeting, with the logistical support of Kiwi Magazine, a publication focused on healthy lifestyle approaches for families. A sample of 1500 parents solicited through Kiwi’s Moms Meet network (https://momsmeet.com, unpublished data) answered questions regarding their knowledge of PIM, PIM therapies they’d like to see offered in pediatric practices, and their willingness to advocate for insurance coverage of PIM therapies. Of note, even in this sample of natural health oriented parents, nearly one in four respondents were not familiar with IM. Fewer than one in seven definitively consider their pediatrician integrative while approximately 60% of parents believe having an integrative pediatrician for their children is important. Information about therapies is most commonly found online, and the two most desired in-office therapies are nutritional counseling and behavioral/mental health services. Key survey findings are shown in Figure 3a–d below.

### 4.3. 2015 PIMLI Summit Summary

At the opening of the 2015 Summit, the 15 PIM leaders in attendance reviewed the professional survey as well as the evolution of the field to date. Using a World Café meeting paradigm, participants explored five strategic domains critical to the sustainable growth of the PIM field: research, clinical practice, professional education, patient and family education, and advocacy and partnerships [23]. Small groups were tasked with describing the existing gaps for each domain with specific strategies to address those gaps. The whole group then worked to clarify gaps and strategies to identify realistic and specific action steps to address each domain.

The following strategies were ultimately identified within each domain:

Research

- Expand the AAP Section on Integrative Medicine research and education grant program.
- Explore the development of a multi-center PIM research network.
- Publish PIM research articles in mainstream academic journals.

Clinical practice

- Promote use of existing quality clinical resources such as *Pediatrics in Review* articles, special issues in professional journals, well-designed research trials on complementary therapies, and integrative pediatrics textbooks.
- Compile and distribute sample encounter forms, handouts, and smart phrases for electronic health records to support PIM clinical care.
- Develop an interprofessional online clinical resource repository.

Professional education

- Maximize distribution and utilization of PIM residency curriculum and fellowship programs.
- Facilitate a regularly occurring interprofessional conference to advance PIM education, leadership development, and to embrace and grow the PIM community.
- Present PIM content at pediatric subspecialty meetings and other health professional conferences, including those targeting the underserved.
- Participate in initiatives to address policy development for modern health problems such as chronic pain and opioid addiction that significantly impact children.

Patient and Family Education

- Develop multilingual descriptions of PIM therapies in child-friendly language.
- Increase PIM content on the AAP’s parent information website (healthychildren.org) by adapting existing *Pediatrics in Review* articles.
- Develop an online video series featuring children and families discussing integrative care success stories.
- Develop a robust library of PIM patient handouts for widespread use in the clinical setting.

Advocacy and Partnerships

- Establish an online presence to serve as a strong independent voice advocating for PIM as mainstream pediatric healthcare, including a repository for research, clinical, and educational resources.
- Partner with disease-specific organizations to spread knowledge of PIM therapies that may improve quality of life for their stakeholders.
- Cultivate strong relationships with specialties also serving children, such as family medicine, medicine/pediatrics, and obstetrics/gynecology.
- Work with federal and state organizations to develop and promote preventive lifestyle policies for children and families.
- Contribute to resource development and advocacy efforts that support payment for PIM services and models of care.
- Explore sustainable resources for philanthropy to support ongoing growth of the field.

The group noted that these are considerable tasks and that an organization does not currently exist to oversee and coordinate all of these strategies. Meeting participants unanimously expressed a strong desire to explore the creation of an ongoing, sustainable PIMLI leadership group to coordinate this work. Immediate next steps were debated, leading to the development of specific action plans related to the above strategies and the delegation to participants. Finally, meeting participants prioritized the following activities most needing financial support:

- Sustainable support for the AAP SOIM research and education grant program.
- Re-printing and redistribution of the “Talk with Your Doctor” PIM poster (http://www.cpsp.cps.ca/uploads/publications/Posters-CAM.pdf) to pediatricians.
- Establishment of a new organization as the independent voice advocating for PIM as mainstream pediatric health care, engaging and nurturing current and future leaders of the PIM movement.

The first two activities were delegated to the AAP SOIM Executive Committee. A PIMLI steering committee was formed to pursue the third priority.

## 5. 2016 PIMLI Summit

With additional generous funding by the Marino Health Foundation, members of the PIMLI steering committee met in July 2016 at the Hackensack University Medical Center in New Jersey to further discuss the establishment of a new organization to coordinate PIM initiatives. In preparation for this meeting, key PIM stakeholders were consulted via an online survey to assess current community needs (unpublished data). Survey participants were recruited from two professional listservs: the IPIM Network and the AAP Section on Integrative Medicine listserv.

### *2016 Professional Survey Highlights* 

- A total 93 health professionals completed the survey.
- Approximately 75% identified as pediatricians (MD/DO) with a total of 17 professional disciplines represented.
- Approximately 50% had been in practice more than 20 years, and more than 80% had been in practice more than 10 years.
- Over 75% stated that currently available PIM resources online are not sufficient to support their pediatric practice.
- The top two most valuable current online PIM resources were the two listservs noted above; no current website was rated as valuable by more than 25% of respondents.

Conversations with key stakeholders and advisors confirmed the need and desire for an independent organization focused specifically on PIM education and advocacy, directed at the broad ‘middle’ of the professional and consumer populations. Challenges identified included: (1) prioritizing PIM both within a larger pediatric organization and within a larger integrative organization; (2) securing funding for administrative support given the significant ongoing clinical, research, and educational commitments of PIM leadership—creating a balanced interprofessional leadership group; and (3) nurturing leadership development to ensure sustainability of the organization. Understanding these obstacles, the PIMLI steering committee evaluated and debated potential partners to host this initiative, concluding that no existing group ideally met our needs. They proposed exploration of hosting an independent nonprofit organization as the first option. Paramount to the success of this organization would be the design and launch of a robust multimedia website that serves as a hub for all PIM activities, including:

- Membership for pediatric healthcare professionals providing specialized access to educational and community-building resources, including integrating existing interprofessional and international PIM networks (IPIM Network, PedCAM network).
- Consumer and policymaker engagement via media tools and the development of short promotional videos profiling PIM “success stories”.
- Educational and Research Resource Repository, including professional presentations and a searchable PIM publication bibliography.
- Continuing education online conferences and webinars.
- Professional consultative service assisting the development of academic PIM centers and clinical practices.
- Coordination of PIM efforts with the AAP SOIM and other reputable national/international organizations.

Organizational structure and funding needs were discussed including potential funding partners and self-sustainable sources of support and leadership. Given the huge scope of the proposed activities above and the clearly expressed needs in the 2016 professional survey, the scope was initially narrowed to online PIM professional education, because opportunities to fill the gap of online educational resources currently exist. At this time, PIM education exists through the University of Arizona Center for Integrative Medicine: Pediatric Integrative Medicine in Residency (PIMR) curriculum for pediatric resident physicians, various continuing education conferences, and online resources [24]. The PIMR program consists of a 100-h online educational curriculum initially piloted in five diverse pediatric residency training programs in the United States. In 2016, the PIMR program had grown to include more than 500 residents nationally and internationally in nine residency programs. Of note, the PIMR program is specifically limited to pediatric resident physicians. The AAP SOIM sponsors PIM educational sessions each year at the AAP National Conference to a broader group of pediatricians, though these sessions are not accessible to many other PIM professional groups nor to nonmembers of the AAP. Ultimately, collaborative efforts to reinforce interactive and online curricula at various steps during training and practice may help to meet the demand for therapeutic options that are safe, effective, and aligned with patients’ values. A comprehensive, accessible online professional PIM education hub would be optimally situated to meet the needs of the larger PIM community.

## 6. Vision for the Future

Based on work at the 2015 and 2016 Summits, long-range visions for PIM’s next decade are outlined:

- PIM exemplifies a mainstream model of comprehensive care that emphasizes a personalized, participatory, and evidence-informed approach to the whole child.
- PIM emphasizes preventive health, understanding of the etiology of underlying illness, and exploration of all appropriate treatment options to optimize health and well-being.
- PIM promotes a cohesive, respectful, and interprofessional team approach focused on the well-being of the child as a growing, developing individual in the context of the family.
- Professionals collaborate across disciplines and geographic boundaries in advocating for the needs of patients and families from all socioeconomic and cultural backgrounds.
- Integrative professionals embrace self-care as a cornerstone of health.
- Integrative professionals support identification of and access to reliable PIM educational, clinical, and research resources for all those interested in maximizing patient health and wellness.

To achieve this vision, proposed tangible outcomes include:

- Establishment of an international and interprofessional pediatric group to coordinate and advance the work of PIM in collaboration with the AAP SOIM. This two-pronged approach ultimately represents the optimal strategy to ensure the sustainable growth, evolution, and impact of PIM within the field of Pediatrics.
- Medical schools, residencies, and other professional training programs teach PIM routinely and widely.
- Hospitals, clinics, and academic centers promote co-located PIM services (e.g., credentialing nurses and/or other providers and staff in biofeedback, aromatherapy, clinical hypnosis, etc.).
- Pediatric training programs implementing the PIMR curriculum and PIM fellowship programs experience high enrollment and active participation of alumni.
- A yearly interprofessional symposium is established and brings together PIM professionals for collaborative learning.
- PIM materials (e.g., description of therapies, licensing/credentialing information, clinical guidance, patient educational materials, research summaries, and updates) are compiled and hosted on an independent website for ready access by professionals, patients, and institutions.
- PIM research is adequately funded to develop the science of utilization, safety, efficacy, and cost-effectiveness of PIM therapies, and a multi-center research network is established to facilitate the acquisition of this knowledge.
- Safe, effective, and cost-effective PIM therapies and health care systems receive endorsement from national organizations, hospital leadership, and other decision makers to judiciously expand PIM as appropriate.
