# Correction: Esparham, A., et al., Pediatric Integrative Medicine: Vision for the Future. *Children*, 2018, *5*, 111

**DOI:** 10.3390/children5090123

**Published:** 2018-09-05

**Authors:** Anna Esparham, Sanghamitra M. Misra, Erica Sibinga, Timothy Culbert, Kathi Kemper, Hilary McClafferty, Sunita Vohra, Lawrence Rosen

**Affiliations:** 1 Division of Child Neurology-Headache Section, Children’s Mercy Hospital, University of Missouri School of Medicine-Kansas City, Kansas City, MO 64108, USA; 2 Mobile Clinic Program, Texas Children’s Hospital, Baylor College of Medicine, Houston, TX 77054, USA; smisra@bcm.edu; 3 Department of Pediatrics, Johns Hopkins School of Medicine, Baltimore, MD 21205, USA; esibinga@jhmi.edu; 4 Integrative Medicine, Prairie Care, University of Minnesota Medical School, Chaska, MN 55318, USA; tculbert@prairie-care.com; 5 Department of Pediatrics, College of Medicine, Ohio State University, Columbus, OH 43210, USA; kathi.kemper@osumc.edu; 6 Department of Medicine, Arizona Center for Integrative Medicine, University of Arizona, Tucson, AZ 85724, USA; hmcclafferty@email.arizona.edu; 7 Integrative Health Institute, CARE Program, PedCAM Network, Department of Pediatrics, Medicine, and Psychiatry, Faculty of Medicine and Dentistry, University of Alberta, Edmonton, AB T6G 2C8, Canada; svohra@ualberta.ca; 8 Whole Child Center, Oradell, NJ 07649, USA; ldrdoc@alum.mit.edu

The authors wish to make the following corrections to their paper [1]:

The middle initial has been added to the name of co-author Sanghamitra Misra. The correct name is Sanghamitra M. Misra.

The citation website link to the first reference in the original paper is now unavailable. The correct reference is as follows:

1. Academic Consortium for Integrative Medicine and Health. Available Online: https://imconsortium.org/about/introduction/ (accessed on 27 August 2018).

In addition, we found that the organization name listed in Section 4 (p. 5) and in the acknowledgments section is incorrect—the reference to the Academic Consortium for Integrative Medicine and Health (ACIHM) is incorrect. The correct organization name is the Academic Collaborative for Integrative Health (ACIH). The corrected last sentence of the second paragraph in Section 4 is as follows:

Specifically, with organizational support by John Weeks, a pioneer in collaborative interprofessional IM efforts over several decades, representatives from the Academic Collaborative for Integrative Health (ACIH) participated in a comprehensive pre-summit survey.

The corrected acknowledgments are as follows:

**Acknowledgments:** The Pediatric Integrative Medicine Leadership Initiative would like foremost to thank the Marino Health Foundation for its generous support of the 2015 and 2016 summits and related work. The PIMLI would also like to acknowledge the American Academy of Pediatrics, in particular Teri Salus and Anne Gramiak, for their logistical support coordinating and facilitating the 2015 PIMLI Summit. PIMLI leaders are extremely grateful to John Weeks and the ACIH committee who helped in preparation for the 2015 summit, and to Kiwi Magazine and the Moms Meet Network for assistance with the 2015 Parent Survey. Finally, the PIMLI group thanks the members of the PIM community who participated in the 2015 and 2016 professional surveys. Thank you to all the PIMLI leaders involved, in addition to the authors: Michelle Bailey, David Becker, Anu French, Scott Shannon, David M. Steinhorn, Minal Vazirani, and Ana Maria Verissimo.

The authors would like to apologize for any inconvenience caused to the readers by these changes that do not affect the scientific results. The manuscript will be updated and the original will remain on the article webpage, with a reference to this Correction.
